# A Systems Pharmacology Approach for Identifying the Multiple Mechanisms of Action of the Wei Pi Xiao Decoction for the Treatment of Gastric Precancerous Lesions

**DOI:** 10.1155/2019/1562707

**Published:** 2019-02-03

**Authors:** Liangjun Yang, Wei Liu, Zhipeng Hu, Maoyi Yang, Jiali Li, Xiangzhen Fan, Huafeng Pan

**Affiliations:** ^1^Pi Wei Institute, Guangzhou University of Chinese Medicine, Guangzhou 510006, China; ^2^Chengdu University of Traditional Chinese Medicine, Chengdu 610075, China; ^3^Pharmacology Institute, Guangzhou University of Chinese Medicine, Guangzhou 510006, China

## Abstract

The Wei Pi Xiao (WPX) decoction, based on the theory of traditional Chinese medicine, has been widely used for the treatment of gastric precancerous lesions (GPL). Although WPX is known to be effective for the treatment of GPL, its active ingredients, cellular targets, and the precise molecular mechanism of action are not known. This study aimed to identify the multiple mechanisms of action of the WPX decoction in the treatment of GPL. The active compounds, drug targets, and the key pathways involved in the therapeutic effect of WPX in the treatment of GPL were analyzed by an integrative analysis pipeline. The information pertaining to the compounds present in WPX and their disease targets was obtained from TCMSP and GeneCards, respectively. The mechanisms underlying the therapeutic effect of WPX were investigated with gene ontology (GO) enrichment analysis and Kyoto Encyclopedia of Genes and Genomes (KEGG) pathway enrichment analysis. A total of 82 bioactive compounds and 146 related targets were identified in this study. Following target analyses, the targets were further mapped to 26 key biological processes and 21 related pathways to construct a target-pathway network and an integrated GPL pathway. The study demonstrated that the WPX formula primarily treats the dysfunctions of GPL arising from cell proliferation, apoptosis, and mucosal inflammation, which offered a novel insight into the pathogenesis of GPL and revealed the molecular mechanism underlying the therapeutic effects of the WPX formula in GPL. This study offers a novel approach for the systematic investigation of the mechanisms of action of herbal medicines, which will provide an impetus to the GPL drug development pipeline.

## 1. Introduction

Gastric cancer is the fifth common malignancy in the world and continues to be the most prevalent cancer in Eastern Asia, especially China [1]. Although several therapeutic strategies have been developed for the treatment of gastric carcinoma over the years, the long-term survival rate is low [2]. It is therefore essential to explore novel molecular targets for the treatment of gastric carcinoma. Gastric carcinogenesis is a multistep and continuous process arising from nonatrophic gastritis, which proceeds to atrophic gastritis, metaplasia, and dysplasia and finally to adenocarcinoma [3]. The high mortality rate of patients with gastric cancer can be reduced by the early detection of precancerous lesions [4]. Therefore, the early intervention of gastric precancerous lesions (GPL), which primarily comprise intestinal metaplasia and dysplasia [5], is an effective strategy for preventing the development of gastric cancer.

In China, traditional Chinese medicine (TCM) has been used for more than 4000 years for the treatment of various diseases [6]. Typically, a combination of plants/minerals is incorporated in the formula, which has immense therapeutic potential for the treatment of complex diseases in a multitargeted manner [7]. The Wei Pi Xiao (WPX) decoction is widely used for the treatment of gastrointestinal diseases, owing to its ability to fortify the spleen, enrich the blood, and dissolve stasis. WPX is a complex TCM prescription comprising 6 different herbs, namely,* Hedysarum multijugum *Maxim* (HMM, Huang-qi), Pseudostellaria heterophylla *(Miq.) Pax* (PHP, Tai-zi-shen), Atractylodes macrocephala *Koidz* (AMK, Bai-zhu), Salvia miltiorrhiza *Bunge* (RMB, Dan-shen), Curcuma zedoaria *(Christm.), Roscoe* (CZR, E-zhu)*, and* Hedyotis diffusa *Willd* (HDW, Bai-hua-she-she-cao). HMM*,* PHP*, and* AMK*, widely used for the treatment of general weakness, have anti-inflammatory and anticarcinogenic activities [8–10] and promote epithelial restitution [11, 12].* RMB* and* CZR* are known to promote blood flow and remove blood stasis according to the theory of TCM, and studies have proven their anti-inflammatory and anticarcinogenic activities [13, 14]. As a traditional heat-clearing and detoxicating herb,* HDW *is frequently used for the treatment of cancers such as gastric cancer, colorectal cancer, and breast cancer by mediating tumor angiogenesis, proliferation, and apoptosis [15–18]. A previous study demonstrated that WPX can favorably reverse gastric intestinal metaplasia and gastric epithelial dysplasia by blocking the Wnt/*β*-catenin pathway [19]. Although the therapeutic efficacy of the WPX formula in the treatment of GPL has been established and the 6 herbs constituting the WPX decoction are known to treat tumors, the active ingredients, the cellular targets, and the precise molecular mechanism(s) of action of WPX are yet to be known.

A new advanced analytical technique called systems pharmacology has been used in TCM research [20], which has received much attention in recent years. By employing a network-based approach, systems pharmacology is able to systematically identify the effect and mechanism of action of medications used for the treatment of complex diseases at the molecular, cellular, tissue, and organismic levels [21]. This research strategy is being extensively applied in recent years for studying numerous TCM formulas such as the Bushen-Yizhi formula and the Qishen-Yiqi dripping pill, and the efficacies of the formulations have been experimentally verified [22, 23].

**Figure 1:**
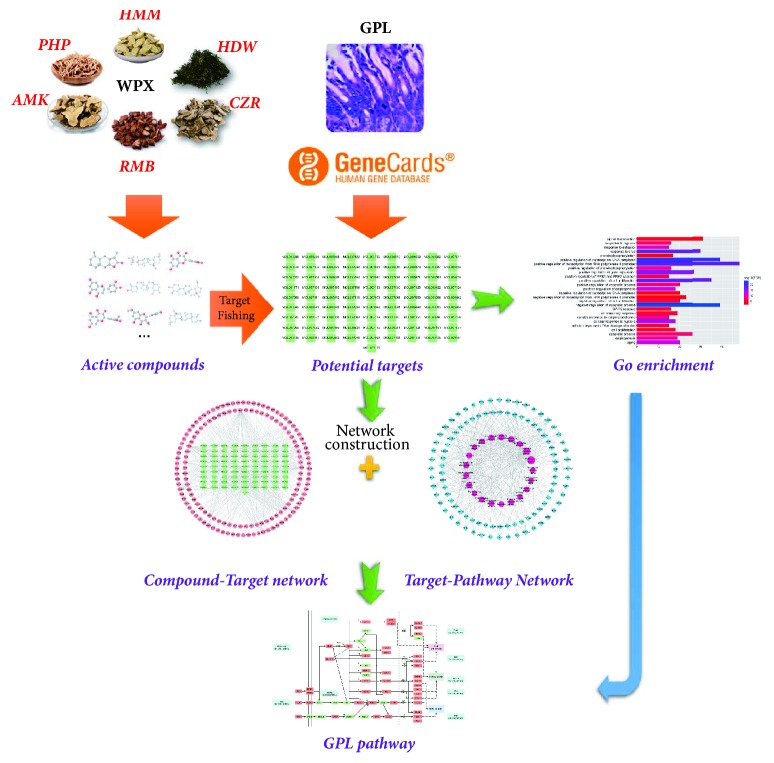
The protocol of the systems pharmacology approach used in this study.

This study attempted to investigate the mechanism of action underlying the therapeutic effect of WPX by employing a systems pharmacology approach. The protocol of the integrated systems pharmacology approach used herein is depicted in Figure 1. The active compounds of WPX were first selected on the basis of their oral bioavailability (OB), drug-likeness (DL), and Caco-2 cell permeability, which were evaluated at the molecular level. The targets were then identified by mapping the drug targets with the therapeutic targets of GPL. The targets thus predicted were further mapped to a compound-target network and validated by gene ontology (GO) enrichment analysis. The targets were subsequently used as baits to fish the corresponding pathways from the Kyoto Encyclopedia of Genes and Genomes (KEGG) database. A target-pathway (T-P) network was constructed for further analyses. Finally, an integrated “GPL pathway” was generated for elucidating the molecular mechanism of action of WPX in the treatment of GPL, which offered a novel approach for furthering understanding of TCM.

## 2. Materials and Methods

### 2.1. Screening Active Compounds

The compounds present in WPX were identified from the Traditional Chinese Medicines for Systems Pharmacology Database and Analysis Platform (TCMSP; http://lsp.nwu.edu.cn/tcmsp.php), which is a database of Chinese herbal medicines providing information on the relationships between drugs, targets, and diseases [24]. The database contains information on 499 herbs and 12144 compounds, obtained through pharmacological studies and clinical knowledge. The compounds present in the WPX decoction were identified from TCMSP. Barring the 55 identical compounds, a total of 432 compounds were identified, which included the 87 compounds present in* HMM*, 25 in* PHP*, 55 in* AMK*, 202 in* RMB*, 81 in* CZR*, and 37 in* HDW*. In order to identify the potential active compounds present in WPX, the compounds were screened on the basis of OB, DL, and Caco-2 cell permeability.

#### 2.1.1. Evaluation of OB

OB is defined as the percentage of unmodified drug that is absorbed into the circulatory system following oral administration. OB is a reliable indicator of the efficacy of an oral administration for drug delivery [25], and bioactive molecules with high OB often have the potential of being developed into drugs [26]. In this study, the OB of the compounds was calculated by the in-house software, OBioavail1.1, which is reasonably capable of accelerating the prediction of OB [27]. Finally, the herbal components having OB ≥ 30% were selected as the candidate molecules for further analyses.

#### 2.1.2. Evaluation of DL

DL is a comparative measure of the functional or physical properties of compounds with those of the majority of known drugs [28]. DL is extensively used for filtering compounds with undesirable properties [29]. Based on the molecular descriptors and the Tanimoto coefficient [30], a self-constructed model was established for calculating the DL index of the compounds in the WPX decoction. The DL was calculated using the following equation:

(1) $$\begin{array}{c} T\left(\;A,B\;\right)=\frac{A\cdot B}{{\left\|A\right\|}^{2}+{\left\|B\right\|}^{2}-A\cdot B} \end{array}$$

where** A** is the molecular property of the herbal compound and** B** represents the average molecular property of all the molecules in the DrugBank database (http://www.drugbank.ca/), which were calculated on the basis of Dragon soft descriptors [31]. The compounds with DL ≥ 0.18 were selected as candidate compounds for subsequent analyses. The threshold value of 0.18 was selected on the basis of the average DL index of all the compounds in DrugBank, which was 0.18.

#### 2.1.3. Evaluation of Caco-2 Cell Permeability

Caco-2 cell permeability is another vital parameter frequently used as a model for studying the passive diffusion of drugs across the intestinal epithelial barrier [32]. In this study, a Caco-2 permeability prediction platform, preCaco-2, was employed for evaluating the drug absorption rate [33], and the baseline Caco-2 cell permeability was set at 0. The threshold values selected for the integrative screening system were OB ≥ 30%, DL ≥ 0.18, and Caco-2 permeability ≥ 0, and the compounds meeting all the three criteria were selected as active compounds for further analyses.

### 2.2. Identifying the Molecular Targets of WPX

Owing to the diversity of the compounds present in the herbal constituents of WPX, the formulation is capable of targeting multiple proteins, thus making target identification a crucial step in understanding the molecular mechanism underlying the therapeutic properties of WPX. In this study, target prediction was achieved using TCMSP, which uses the SysDT model and the Herb Ingredients' Targets (HIT) database [24]. The SysDT model is developed from two powerful methods, Random Forest (RF) and Support Vector Machine (SVM), and the performance of this model in predicting drug-target interactions is outstanding, with a concordance of 82.83%, a sensitivity of 81.33%, and a specificity of 93.62% [34]. In instances where the targets could not be identified, the Swiss Target Prediction database was used. The Swiss Target Prediction database allows the target prediction of bioactive small molecules based on a combination of 2D and 3D similarity measures with known ligands [35]. In this study, molecular information was retrieved in the form of simplified molecular input line entry specification (SMILES) or Structure from PubChem (https://pubchem.ncbi.nlm.nih.gov/), which is an open chemistry database containing information on the chemical structures, identifiers, chemical and physical properties, biological activities, and other molecular properties of compounds. The compounds were subsequently submitted to the Swiss Target Prediction database for identifying the potential targets, and the confidence score of the prediction probability of a target protein was more than 40%.

Owing to the noncanonical description of the targets thus identified, the UniProt Knowledgebase (UniProtKB; www.uniprot.org/) was used. The identified candidate targets were treated as the query, and the hits categorized under ‘*Homo sapiens*' were selected from UniProtKB. The molecular targets of the compounds of WPX, along with their gene symbols, were retrieved from the database.

### 2.3. Disease Targets of GPL

The genes associated with GPL were identified from the Human Gene Database, known as GeneCards (http://www.genecards.org/), which is a searchable, integrative, user-friendly database that provides comprehensive information on all predicted and annotated human genes, proteins, and diseases [36]. The GeneCards database comprises information from 125 different databases such as HGNC, NCBI, ENSEMBL, and UniProtKB, apart from numerous other related databases, and the information content is considered to be reliable [37]. In order to retrieve information about the related targets from the database, a keyword-based search was performed using the keywords ‘gastric precancerous lesions' or ‘precancerous lesion of gastric cancer'.

### 2.4. GO Analysis and KEGG Pathway Enrichment

The GO defines concepts related to gene function and the interrelationships among the functions of different genes. It describes the functions of herbal components in terms of the molecular function(s), the cellular component(s) involved, and the biological process affected [38]. In this study, a GO analysis was performed for understanding the concerned biological processes. KEGG pathway enrichment was additionally performed, for studying the biological effects of the WPX decoction at the pathway level. GO analysis and pathway enrichment were conducted by linking the targets to DAVID (database for annotation, visualization, and integrated discovery; http://david.abcc.ncifcrf.gov). The enriched GO terms and pathways having a false discovery rate (FDR) of less than 0.01 according to Fisher's exact test were selected and subjected to further analyses.

### 2.5. Network Construction

In order to elucidate the molecular mechanisms underlying the complex therapeutic property of WPX in the treatment of GPL, a compound-target network (C-T network) and a T-P network were generated for studying the relationships among the candidate compounds and the potential disease targets. (1) C-T network: The compound-target interactions were visualized by the C-T network, in which all the active ingredients were connected to their corresponding targets. (2) T-P network: The potential targets identified by preliminary analyses were mapped to the DAVID database for conducting pathway enrichment. The relationships between these potential pathways and GPL were subsequently elucidated by literature mining.

The bipartite graphs were visualized and analyzed by Cytoscape version 3.2.1 [39], which is a robust software for data visualization and integration in Bioinformatics. In this network, the nodes represented the drug compounds and targets, while the edges represented the interactions between them. On the other hand, a vital topological parameter, named degree, was analyzed by the Network Analyzer plugin of Cytoscape [40]. The degree of a node referred to the number of edges connected to the node.

### 2.6. Construction of the GPL Pathway

For a better understanding of the mechanisms underlying the therapeutic effects of WPX against GPL, an integrated “GPL pathway” was manually constructed based on the T-P network previously generated. The pathways in the T-P network which had no direct and close connections with the disease were removed.

## 3. Results

### 3.1. Screening Active Compounds

In order to identify the active ingredients in the WPX decoction, three classical ADME parameters, namely OB, DL, and Caco-2 cell permeability, were employed for screening the compounds. Following screening, 88 potential active compounds, representing 20.37% of the total number of compounds present in WPX, were identified, which included 16 compounds from* HMM*, 7 from* PHP*, 7 from* AMK*, 54 from* RMB*, 3 from* CZR*, and 7 from* HDW*. Since it was possible that the compounds which did not satisfy the screening criteria could also have therapeutic effects in humans, certain compounds were retained as active components on the basis of available information pertaining to their pharmaceutical activities, even if they did not match the screening criteria. For instance, although astragaloside IV (MOL000407, OB = 22.50, DL = 0.15, and Caco-2 permeability = -2.11) has poor OB, DL, and Caco-2 permeability, it was retained as an active compound since it is the major constituent of* HMM *[41]. Studies have demonstrated that astragaloside IV induces anti-inflammatory effects in gastric tissues by suppressing the expression of inflammatory cytokines, such as TNF-*α* and IL-1*β* [42], and restrains epithelial-mesenchymal transitions by inhibiting the PI3K/AKT/NF- kappa B pathway in gastric cancer cells [43]. Additionally, a bioactive constituent of* RMB*, known as danshensu (MOL007134, OB = 36.91, DL = 0.06, and Caco-2 permeability = -0.27) is a potential antithrombotic and antiplatelet agent, owing to its highly selective inhibition of COX-2 [44]. Atractylenolide I, curcumol, and oleanolic acid were similarly retained as active compounds although they did not satisfy the screening criteria. These 5 compounds of WPX were additionally retained as active compounds. In conclusion, a total of 88 compounds were identified from the WPX decoction on the basis of their biological activities, and a total of 93 compounds were selected as active herbal constituents in this study (represented in Table S1).  Table 1 showed parts of compounds from WPX and their corresponding predicted OB, DL, and Caco-2 scores and structure.

### 3.2. Target Identification and Analysis

Based on the aforementioned target fishing approach, a total of 306 targets were predicted to interact with the 93 compounds identified from the 6 herbs in the WPX decoction. However, the targets specific to GPL as well as their mechanisms of interaction were not known. In order to validate the relevance of these proteins in the development of GPL, the GeneCards database was employed for identifying the disease-related genes. Since GeneCards is a gene-centric database, the disease-associated genes are presented in an integrated web card, representing nearly 90% of the human protein-coding genes [45]. A total of 1261 GPL-related genes were identified from the database by employing a keyword-based search. Upon combining the compound targets of WPX with the disease targets, a total of 146 overlapping genes were selected as the potential targets for the treatment of GPL. By this process, 82 compounds were ultimately selected as the active herbal ingredients after discarding 11 candidate compounds that had no relevant targets. Since the mechanisms underlying the therapeutic effects of TCM formulas are due to the synergistic effects of multiple compounds and targets [46], and since the pivotal targets are the cores of the network from the point of network topology [47], the 146 genes identified herein were considered to be effective therapeutic targets (refer to Table S2).

**Figure 2:**
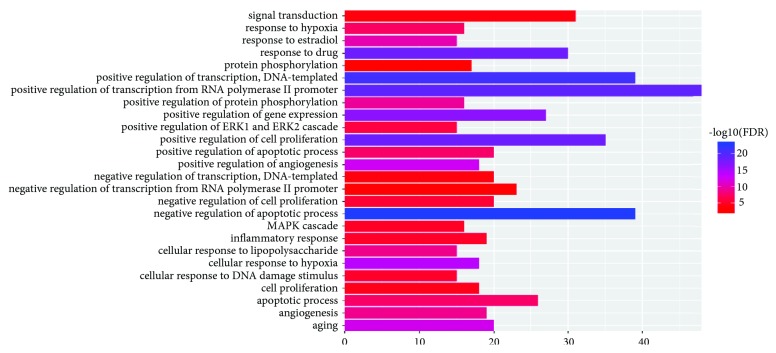
Gene ontology (GO) analysis of the target genes associated with GPL. The X-axis represents the significant enrichment counts of these terms, while the Y-axis represents the categories of ‘biological process' in the GO of the target genes (FDR ≤ 0.01).

In order to validate whether the 146 targets are associated with GPL, a GO analysis was performed for elucidating the concerned biological processes.  Figure 2 represents the first 26 significantly enriched GO terms (FDR ≤ 0.01) for these targets. The* p*-values, FDR, and counts are provided in Table S3. The results indicated that numerous targets are involved in the process of tumorigenesis, including those involved in positive transcriptional regulation by RNA polymerase II promoter (GO:0045944), negative regulation of apoptosis process (GO:0043066), apoptotic process (GO:0006915), positive regulation of cell proliferation (GO:0008284), and inflammatory response (GO:0006954).

### 3.3. Network Construction and Analysis

#### 3.3.1. Construction and Analysis of the C-T Network

**Figure 3:**
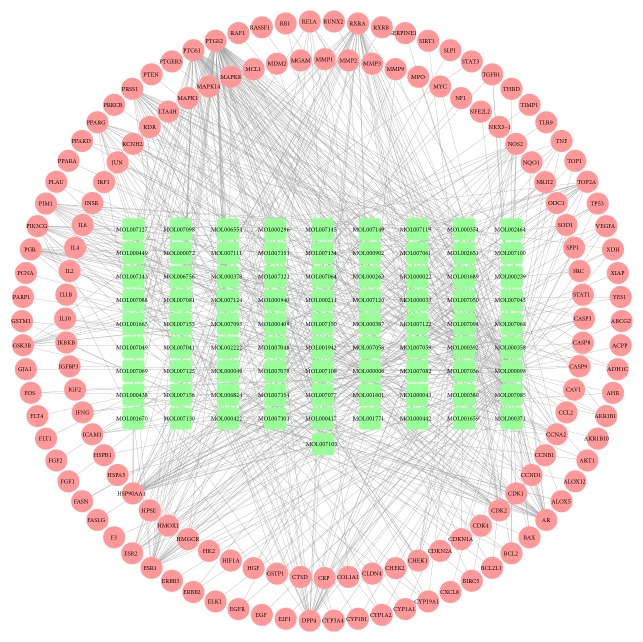
The C-T network generated in this study. The red nodes represent the potential targets, and the green nodes represent the herbal compounds, while the lines represent the interactions between them.

In order to understand the relationships among the herbal constituents in the WPX decoction, the compound targets, and the GPL targets, a C-T network was constructed. The active compounds, targets, and the interactions among them are represented in Figure 3, with 228 nodes, representing the 82 potential compounds and 146 potential targets, and 677 edges. The yellow and green nodes represent the targets and the compounds, while the edges represent the interactions between them. In general, the degree of target interactions is an indicator of the potential significance of the compounds. Analysis of the C-T network revealed that for each compound the average degree was 4.64, and the average number of edges was 8.26, indicating that the compounds with a high degree might be crucial for the treatment of GPL.

#### 3.3.2. Construction and Analysis of the T-P Network

**Figure 4:**
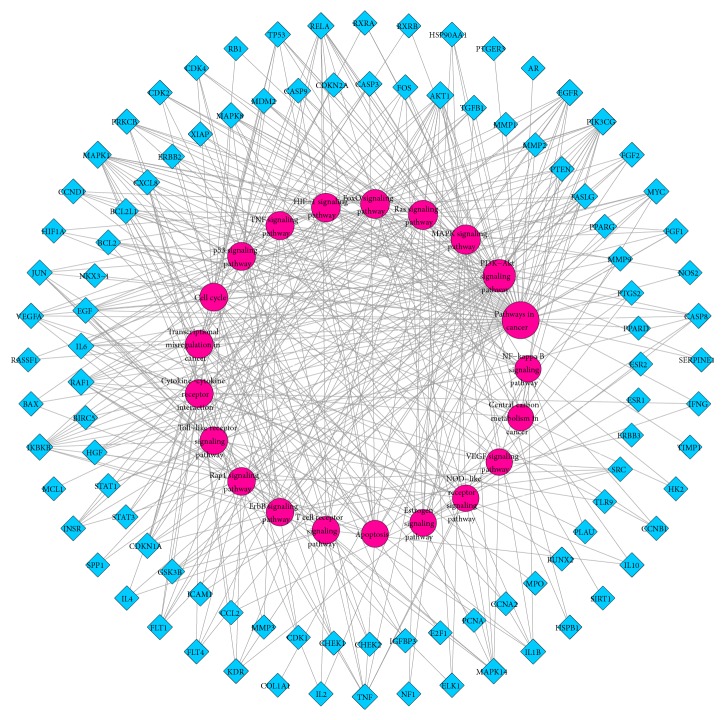
The T-P network generated in this study. The blue nodes represent potential targets and the red nodes represent the related pathways. The sizes of the nodes are in proportion to their degree.

In this study, 146 targets were mapped to 115 pathways following KEGG pathway enrichment. After combining the pathological data obtained from literature mining with the FDR scores (FDR ≤ 0.01), the pathways that had no relationships with GPL were discarded. Finally, 21 remarkably enriched pathways that were likely to be the major pathways in the treatment of GPL were selected (refer Table S4). The T-P network was subsequently generated by mapping 97 of the 146 targets to the major target pathways (Figure 4).

As demonstrated in Figure 4, these targets closely interacted with the pathways involved in cancer (hsa05200, degree = 57), the PI3K-Akt signaling pathway (hsa04151, degree = 37), the MAPK signaling pathway (hsa04010, degree = 24), and the Ras signaling pathway (hsa04014, degree = 23), among others. These pathways are regarded as the key pathways responsible for the progression of GPL.

### 3.4. The GPL Pathway

**Figure 5:**
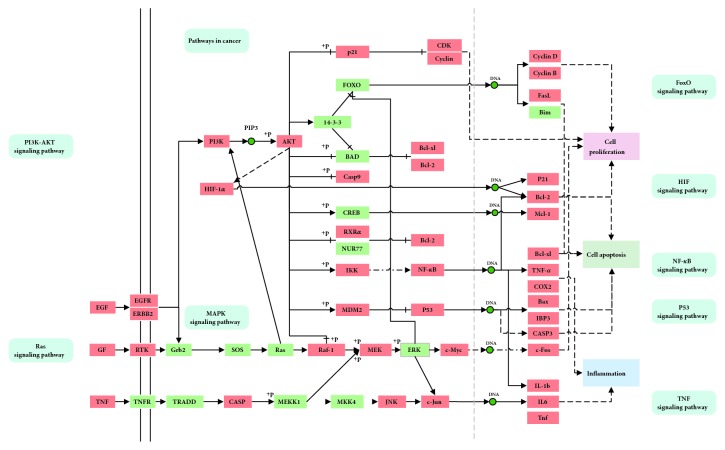
The GPL pathway constructed in this study. The orange nodes represent the potential disease protein targets, while the green nodes represent the relevant targets in the pathway.

Based on the aforementioned results, an integrated “GPL pathway” was constructed by integrating the key pathways, which included the pathways involved in cancer (hsa05200), the PI3K-Akt signaling pathway (hsa04151), the MAPK signaling pathway (hsa04010), the Ras signaling pathway (hsa04014), the FoxO signaling pathway (hsa04068), the HIF-1 signaling pathway (hsa04066), the TNF signaling pathway (hsa04668), the p53 signaling pathway (hsa04115), and the NF-kappa B signaling pathway (hsa04064). As shown in Figure 5, the GPL pathway can be separated into three representative therapeutic modules, namely, the cell proliferation module, the cell apoptosis module, and the inflammation module.

#### 3.4.1. The Cell Proliferation Module

The disruption in the balance between the proliferation and apoptosis of gastric epithelial cells is linked to the progression from chronic gastritis, to atrophy, intestinal metaplasia, dysplasia, to ultimately cancer [48]. As demonstrated in Figure 5, the PI3K-Akt and MAPK signaling pathways are closely related to cell proliferation and play an important role in gastric tumourigenesis [49–51]. For instance, the serine/threonine protein kinase, Akt, is involved in regulating a plethora of cellular processes triggered by a wide diversity of extracellular signals and is thus considered to be a key molecule in the PI3K-Akt signaling pathway [52]. Certain compounds in WPX, such as quercetin (MOL000098) from* HMM *and* HDW* and luteolin (MOL000006) from* PHP* and* RMB*, have been shown to be effective in downregulating the phosphorylation of Akt, which leads to the inhibition of cell proliferation [53, 54]. Additionally, tanshinone IIA (MOL007154), kaempferol (MOL000422), and beta-sitosterol (MOL000358) target JUN, which ultimately interferes with the MAPK signaling pathway, thus inducing apoptosis [55–57]. All these data indicate that the therapeutic effect of WPX in treating GPL could be mediated by regulating cell proliferation.

#### 3.4.2. The Cell Apoptosis Module

Apoptosis is a crucial mechanism leading to cell death, and the failure to inhibit apoptosis induces the formation of certain gastrointestinal malignancies [58]. It has been further demonstrated that apoptosis plays a vital role in the morphogenesis of GPL [59]. As shown in Figure 5, certain targets in the p53 signaling pathway, the TNF signaling pathway, the Ras signaling pathway, and the FoxO signaling pathway are involved in the process of necrocytosis. For instance, caspase 3 (CASP3), a member of the interleukin-1 beta-converting enzyme family that participates in the TNF and p53 pathways, induces apoptosis [60] and is identified as one of the key effector caspases in the apoptotic machinery [61]. Our results also demonstrated that CASP3 can be regulated by acacetin (MOL001689) from* PHP*, oleanolic acid (MOL000263) from* RMB *and* HDW*, and kaempferol (MOL000422) from* HMM.* In the Ras and FoxO pathways, the Fas ligand (FasL) that belongs to the TNF family leads to apoptosis upon binding to its receptor and significantly influences the progression of cancer [62]. The aforementioned observations suggest that the therapeutic effect of WPX in the treatment of GPL could be mediated by the induction of apoptosis in the epithelial cells of the gastric mucosa.

#### 3.4.3. The Inflammation Module

Chronic mucosal inflammation is associated with a high risk of progression from chronic gastritis to gastric cancer [63]. Chronic inflammation following infection with* Helicobacter pylori* is recognized as a risk factor for atrophic gastritis, intestinal metaplasia, and adenocarcinoma [64]. As indicated in Figure 5, the inflammatory cytokines including TNF, IL1, IL6, and COX-2 are involved in the TNF signaling pathway, the NF-kappa B signaling pathway, and the MAPK signaling pathway. For instance, COX-2, which is known to induce inflammation and cause tumorigenesis via the NF- kappa B pathway, also participates in the invasion and metastasis of cancer cells [65]. A study demonstrated that danshensu (MOL007134), which is obtained from* RMB*, has COX-2-dependent anticancer properties [66]. These data suggest that the agents that regulate cytokines can suppress inflammation and thereby inhibit the progression of GPL.

## 4. Discussion

Gastric cancer is one of the most common types of cancer, and the mortality rate has markedly improved [67, 68]. Early diagnosis and treatment of GPL are crucial to reduce the morbidity and mortality of gastric cancer [5, 69]. However, there remain some medical controversies of GPL not solved satisfactorily with current western allopathic therapy[70]. As a useful alternate medicine, TCM is attracting more and more attention across the world for its remarkable effects in clinical practice. WPX, a Chinese herb formula, has been used to treat GPL effectively. More and more evidence shows that herbs and their active compounds in this decoction have biological effects on GPL. Thus, it is imperative to use the systems pharmacology approach combining the screening active components, drug targeting, network, and pathway analysis to explore the therapeutic mechanism of WPX in the treatment of GPL.

The results show that 82 active compounds were obtained from WPX, and 146 potential targets were found to be linked to multiple compounds from different herbs. These indicate that WPX exerts therapeutic effects on GPL through multiple compounds and targets. Among the active compounds linked to the network, quercetin (MOL000098, degree = 104), luteolin (MOL000006, degree = 46), kaempferol (MOL000422, degree = 37), isorhamnetin (MOL000354, degree = 37), acacetin (MOL001689, degree = 20), and astragaloside IV (MOL000409, degree = 4) are well-known bioactive compounds in the treatment of gastric cancer [71–76]. For instance, the compound quercetin from* HMM* and* HDW* exhibited the highest degree number of interactions with various protein targets. Being the main dietary flavonoid, quercetin not only functions as a radical-scavenging antioxidant, but also suppresses inflammation for inhibiting carcinogenesis [77]. According to a population-based study, the dietary intake of quercetin can reduce the incidences of stomach cancer, particularly in women who have been exposed to tobacco smoke [78]. Furthermore, the antitumor effect of quercetin is associated with the activation of autophagy via modulation of the signaling pathways mediated by AKT-mTOR and hypoxia-induced factor 1*α* (HIF-1*α*) [79].

On the other hand, numerous targets were found to be linked to multiple compounds from different herbs, which indicated the synergistic property of the compounds in the WPX decoction in the treatment of GPL. The target PTGS2 (Prostaglandin-Endoperoxide Synthase 2) for instance, also known as COX-2, was connected to 64 active compounds of the WPX formula. COX-2 plays an important role in the progression of GPL [80], and a reduction in the levels of COX-2 is related to the regression of precancerous lesions [81]. It has been revealed that celecoxib, a selective COX-2 inhibitor, has therapeutic effects on the regression of advanced gastric lesions [82]. By analyzing the constituents and targets, the network revealed that the WPX formula may treat GPL via numerous pathways and cellular processes. According to GO enrichment and KEGG pathway enrichment analyses, we infer that WPX may exert a therapeutic effect by interfering with apoptosis and cell proliferation, and mucosal inflammation. For instance, the GO term like RNA polymerase II, one of the RNAP enzymes found in the nucleus of eukaryotic cells [83], is associated with the survival of gastric cancer cells [84]. It has been additionally confirmed that other GO terms such as negative regulation of apoptosis process, apoptotic process, and positive regulation of cell proliferation are closely associated with gastric carcinogenesis [85]. Among the enriched pathways linked to the network, the PI3K-Akt signaling pathway could sense cell growth factors and is frequently activated by genome amplification in gastric cancer [86]. The MAPK signaling pathway, which was first discovered in cancer cells, plays an important role in transducing the external signals from mitogens into intracellular signaling events that promote cell growth, proliferation, and differentiation [87] and acts as a primary mediator of inflammation during tumor progression [88]. Some pathways such as the TNF signaling pathway (hsa04668, degree = 19), the p53 signaling pathway (hsa04115, degree = 18), and pathways involved in cell cycle progression (hsa04110, degree = 18) and apoptosis (hsa04210, degree = 14) have been established as target pathways for the treatment of GPL. Our previous study demonstrated that WPX can suppress the progression of GPL by inducing apoptosis via stimulation of p53 expression [89]. Additionally, certain pathways related to cell proliferation and apoptosis such as the Ras signaling pathway (hsa04014, degree = 23), the VEGF signaling pathway (hsa04370, degree = 12), and the NF-kappa B signaling pathway (hsa04064, degree = 11) have been established as potential target pathways for the treatment of gastric carcinogenesis [90]. It is well known that inflammation plays decisive role at different stages of tumor development, including initiation, promotion, malignant conversion, invasion, and metastasis[91]. WPX can reduce inflammation and reverse the progression of GPL by regulating the expression of TNF[92], which involves in the MAPK signaling pathway, TNF signaling pathway, NOD-like receptor signaling pathway, and NF-kappa B signaling pathway. On the basis of the results obtained in this study, we speculate that the WPX decoction targets multiple signaling pathways in an integrated manner to regulate cell proliferation, evade apoptosis, and suppress inflammation.

Although numerous researches have demonstrated that TCM formulations are effective and safe in treating GPL [93], the mechanisms of action are not fully revealed. It is imperative to illustrate the molecular mechanism of WPX in GPL treatment. The result indicated that the pharmacological mechanisms of WPX on GPL might be strongly associated with its synergic modulation on cell proliferation, cell apoptosis, and inflammation. The systems pharmacology approach applied in this work provides a novel way to decipher the underlying mechanisms of WPX and contribute to a better understanding of herbal medicines. However, there was no experimental verification in this work, such as quantitative real-time PCR or western blot analysis to estimate the predictions. Further works are required to confirm the results of this study and to reveal the key mechanisms.

## 5. Conclusions

GPL is related to the development of gastric cancer that progressively advances through the formation of sequential lesions, and the treatment of precancerous lesions offers an effective measure for decreasing morbidity in the initial phase of gastric carcinogenesis. In this study, we employed a systems pharmacology approach by integrating active compound screening, target prediction, network analysis, and pathway analysis to explore the molecular mechanisms underlying the therapeutic effect of the WPX decoction in the treatment of GPL. The key results of this study are as follows:

1. A total of 82 bioactive compounds were identified from WPX, which provided potential clues for investigating the molecular mechanism underlying the therapeutic effect of WPX.
2. A total of 146 targets were predicted by comparing the targets of the WPX compounds with the GPL targets, which further demonstrated the characteristic multitargeting property of WPX. The results of GO enrichment and C-T network analysis revealed that WPX exerts a therapeutic effect by interfering with apoptosis and cell proliferation.
3. The results of GO enrichment and the integrated GPL pathways revealed that the WPX decoction targets 26 key biological processes and 21 pathways involved in the pathogenesis of GPL, which further demonstrated the three dysfunctions of GPL, namely, cell proliferation, apoptosis, and mucosal inflammation.
4. On the whole, this study investigated the mechanisms of action of WPX in the treatment of GPL and provides a novel approach for exploring TCM formulations. The results will advance the cognitive understanding of TCM formulations and promote the therapeutic application of traditional medicines for the treatment of diseases in the present day scenario.

## Figures and Tables

**Table 1:** 24 representative components from WPX and their corresponding predicted OB, DL, Caco-2 scores and structure.

**No.**	**Mol ID**	**Molecule name**	**OB**	**DL**	**Caco-2**	**Structure**	**Herb**
1	MOL000006	Luteolin	36.16	0.25	0.19	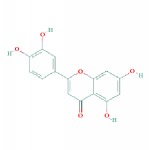	*PHP, RMB*
2	MOL000043	Atractylenolide I	37.37	0.15	1.30	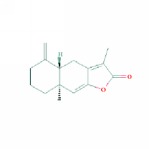	*AMK*
3	MOL000098	Quercetin	46.43	0.28	0.05	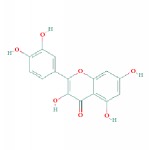	*HMM, HDW*
4	MOL000211	Mairin	55.38	0.78	0.73	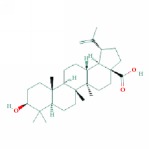	*HMM*
5	MOL000239	Jaranol	50.83	0.29	0.61	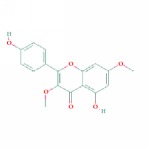	*HMM*
6	MOL000263	Oleanolic acid	29.02	0.76	0.59	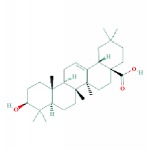	*RMB, HDW*
7	MOL000296	Hederagenin	36.91	0.75	1.32	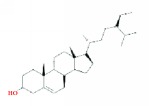	*HMM, CZR*
8	MOL000354	Isorhamnetin	49.60	0.31	0.31	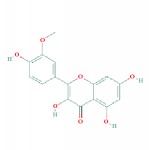	*HMM*
9	MOL000358	Beta-sitosterol	36.91	0.75	1.32	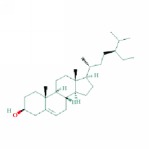	*PHP, HDW*
10	MOL000409	Astragaloside IV	17.74	0.15	(2.22)	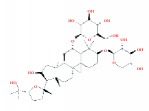	*HMM*
11	MOL000417	Calycosin	47.75	0.24	0.52	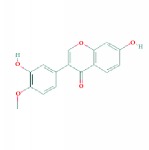	*HMM*
12	MOL000422	kaempferol	41.88	0.24	0.26	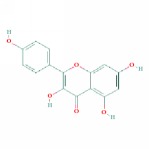	*HMM*
13	MOL000449	Stigmasterol	43.83	0.76	1.44	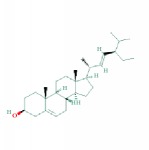	*HDW*
14	MOL000902	Curcumol	103.55	0.13	1.12	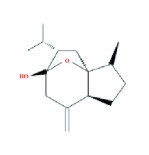	*CZR*
15	MOL000906	Wenjine	47.93	0.27	0.30	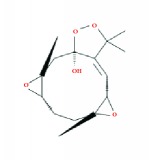	*CZR*
16	MOL001659	Poriferasterol	43.83	0.76	1.44	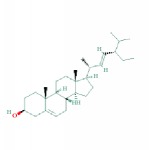	*RMB, HDW*
17	MOL001689	Acacetin	34.97	0.24	0.67	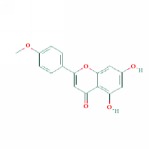	*PHP*
18	MOL006554	Taraxerol	38.40	0.77	1.37	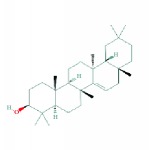	*PHP*
19	MOL006756	Schottenol	37.42	0.75	1.33	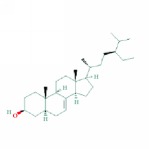	*PHP*
20	MOL007111	Isotanshinone II	49.92	0.40	1.03	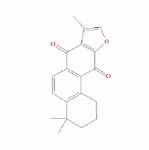	*RMB*
21	MOL007134	Danshensu	36.91	0.06	-0.27	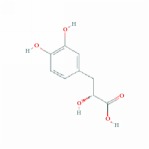	*RMB*
22	MOL007151	Tanshindiol B	42.67	0.45	0.05	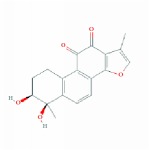	*RMB*
23	MOL007154	Tanshinone IIA	49.89	0.40	1.05	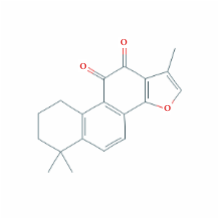	*RMB*
24	MOL007156	Tanshinone VI	45.64	0.30	0.48	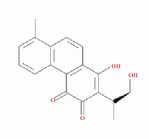	*RMB*

## Data Availability

The compounds information from WPX, the targets information about GPL, the GO terms, and the KEGG pathways used to support the findings of this study are included within the supplementary information files.

## Supplementary Materials

Supplementary Materials Table S1: 93 compounds from WPX and their corresponding predicted OB, DL, and Caco-2 scores and structures. 88 compounds that meet the parameters OB ≥ 30%, DL ≥ 0.18, and Caco-2 ≥0 were preserved as active compounds. 5 compounds which do not meet the criterion but have been validated with various pharmaceutical activities were also reserved as the active components. Table S2: The information of GPL-related targets. By combining the compound targets of WPX and the disease-related targets, 146 overlapping ones were selected as the key targets in the treatment of GPL. Table S3: The GO terms of therapy target genes and their corresponding count,* p*-values, and FDR. Through the GO enrichment of the key targets, 26 top GO terms were obtained which indicate that large numbers of targets were involved in the process of tumorigenesis. Table S4: The KEGG pathways of therapy target genes and their corresponding count,* p*-values, and FDR. Through the KEGG enrichment of the key targets, 21 remarkably enriched pathways involved in cell proliferation, apoptosis, and inflammation were obtained.
