# Supplementary material for: A Systems Pharmacology Approach for Identifying the Multiple Mechanisms of Action of the Wei Pi Xiao Decoction for the Treatment of Gastric Precancerous Lesions
Source: Evid Based Complement Alternat Med. 2019 Feb 3;2019:1562707. doi: 10.1155/2019/1562707 (PMC6378068; doi:10.1155/2019/1562707)
Supplement: Supplementary Materials — Table S1: 93 compounds from WPX and their corresponding predicted OB, DL, and Caco-2 scores and structures. 88 compounds that meet the parameters OB ≥ 30%, DL ≥ 0.18, and Caco-2 ≥0 were preserved as active compounds. 5 compounds which do not meet the criterion but have been validated with various pharmaceutical activities were also reserved as the active components. Table S2: The information of GPL-related targets. By combining the compound targets of WPX and the disease-related targets, 146 overlapping ones were selected as the key targets in the treatment of GPL. Table S3: The GO terms of therapy target genes and their corresponding count, p-values, and FDR. Through the GO enrichment of the key targets, 26 top GO terms were obtained which indicate that large numbers of targets were involved in the process of tumorigenesis. Table S4: The KEGG pathways of therapy target genes and their corresponding count, p-values, and FDR. Through the KEGG enrichment of the key targets, 21 remarkably enriched pathways involved in cell proliferation, apoptosis, and inflammation were obtained. [file 1562707.f1.docx]

**Table S1**

93 compounds from WPX and their corresponding predicted OB, DL, Caco-2 scores and structures. 88 compounds that meet the parameters which OB ≥ 30%, DL ≥ 0.18, Caco-2 ≥0 were preserved as active compounds. 5 compounds which not meet the criterion but have been validated with various pharmaceutical activities were also reserved as the active components.

| **No.** | **Mol ID** | **Molecule name** | **OB** | **DL** | **Caco-2** | **Structure** | **Herb** |
| --- | --- | --- | --- | --- | --- | --- | --- |
| 1 | MOL000006 | Luteolin | 36.16 | 0.25 | 0.19 | 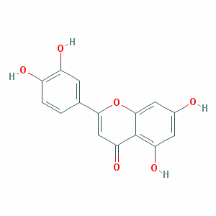 | *PR，RS* |
| 2 | MOL000020 | 12-senecioyl-2E,8E,10E-atractylentriol | 62.40 | 0.22 | 0.01 | 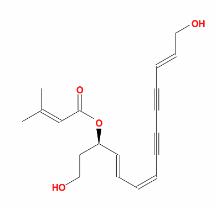 | *AMK* |
| 3 | MOL000021 | 14-acetyl-12-senecioyl-2E,8E,10E-atractylentriol | 60.31 | 0.31 | 0.33 | 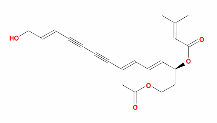 | *AMK* |
| 4 | MOL000022 | 14-acetyl-12-senecioyl-2E,8Z,10E-atractylentriol | 63.37 | 0.30 | 0.42 | 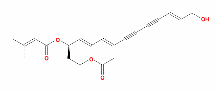 | *AMK* |
| 5 | MOL000028 | α-Amyrin | 39.51 | 0.76 | 1.42 | 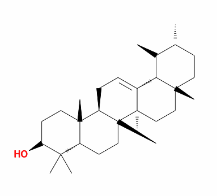 | *AMK* |
| 6 | MOL000033 | (3S,8S,9S,10R,13R,14S,17R)-10,13-dimethyl-17-[(2R,5S)-5-propan-2-yloctan-2-yl]-2,3,4,7,8,9,11,12,14,15,16,17-dodecahydro-1H-cyclopenta[a]phenanthren-3-ol | 36.23 | 0.78 | 1.45 | 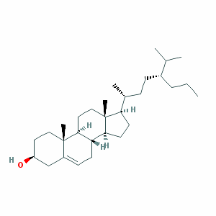 | *HMM，AMK* |
| 7 | MOL000043 | AtractylenolideⅠ | 37.37 | 0.15 | 1.30 | 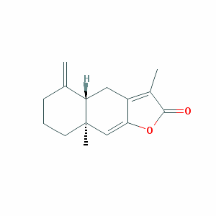 | *AMK* |
| 8 | MOL000049 | 3β-acetoxyatractylone | 54.07 | 0.22 | 1.13 | 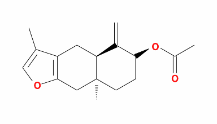 | *AMK* |
| 9 | MOL000072 | 8β-ethoxy atractylenolide Ⅲ | 35.95 | 0.21 | 1.08 | 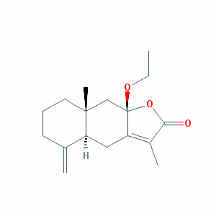 | *AMK* |
| 10 | MOL000098 | Quercetin | 46.43 | 0.28 | 0.05 | 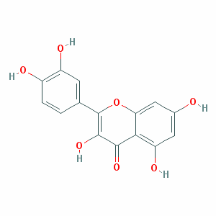 | *HMM，HDH* |
| 11 | MOL000211 | Mairin | 55.38 | 0.78 | 0.73 | 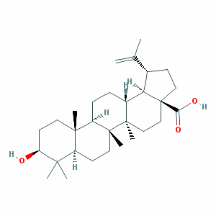 | *HMM* |
| 12 | MOL000239 | Jaranol | 50.83 | 0.29 | 0.61 | 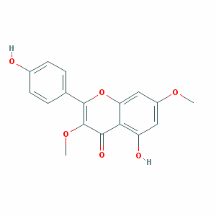 | *HMM* |
| 13 | MOL000263 | Oleanolic acid | 29.02 | 0.76 | 0.59 | 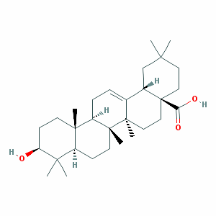 | *RS，HDH* |
| 14 | MOL000296 | Hederagenin | 36.91 | 0.75 | 1.32 | 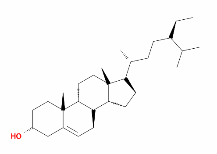 | *HMM，CR* |
| 15 | MOL000354 | Isorhamnetin | 49.60 | 0.31 | 0.31 | 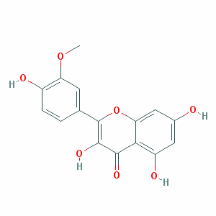 | *HMM* |
| 16 | MOL000358 | Beta-sitosterol | 36.91 | 0.75 | 1.32 | 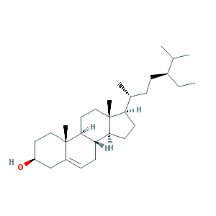 | *PR，HDH* |
| 17 | MOL000371 | 3,9-di-O-methylnissolin | 53.74 | 0.48 | 1.18 | 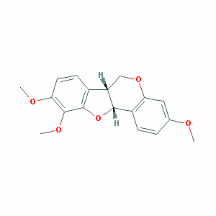 | *HMM* |
| 18 | MOL000378 | 7-O-methylisomucronulatol | 74.69 | 0.30 | 1.08 | 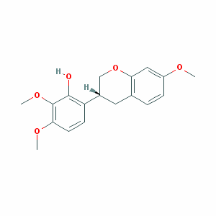 | *HMM* |
| 19 | MOL000380 | (6aR,11aR)-9,10-dimethoxy-6a,11a-dihydro-6H-benzofurano[3,2-c]chromen-3-ol | 64.26 | 0.42 | 0.93 | 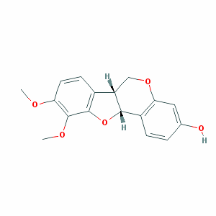 | *HMM* |
| 20 | MOL000387 | Bifendate | 31.10 | 0.67 | 0.15 | 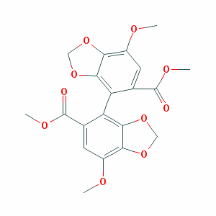 | *HMM* |
| 21 | MOL000392 | Formononetin | 69.67 | 0.21 | 0.78 | 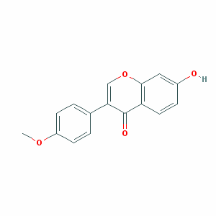 | *HMM* |
| 22 | MOL000398 | Isoflavanone | 109.99 | 0.30 | 0.53 | 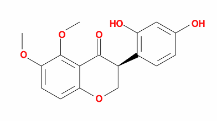 | *HMM* |
| 23 | MOL000409 | Astragaloside IV | 17.74 | 0.15 | (2.22) | 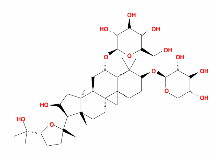 | *HMM* |
| 24 | MOL000417 | Calycosin | 47.75 | 0.24 | 0.52 | 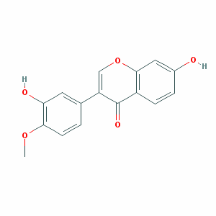 | *HMM* |
| 25 | MOL000422 | kaempferol | 41.88 | 0.24 | 0.26 | 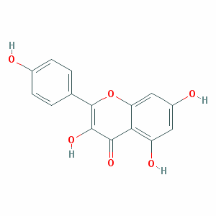 | *HMM* |
| 26 | MOL000438 | (3R)-3-(2-hydroxy-3,4-dimethoxyphenyl)chroman-7-ol | 67.67 | 0.26 | 0.96 | 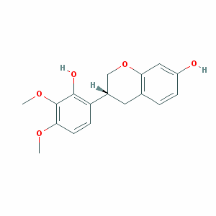 | *HMM* |
| 27 | MOL000442 | 1,7-Dihydroxy-3,9-dimethoxy pterocarpene | 39.05 | 0.48 | 0.89 | 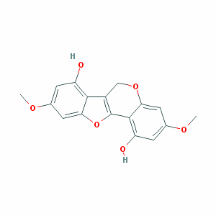 | *HMM* |
| 28 | MOL000449 | Stigmasterol | 43.83 | 0.76 | 1.44 | 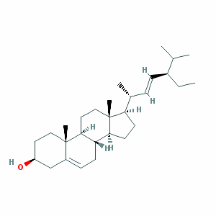 | *HDH* |
| 29 | MOL000902 | Curcumol | 103.55 | 0.13 | 1.12 | 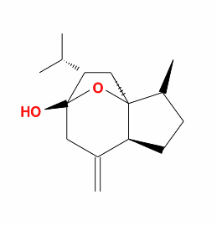 | *CR* |
| 30 | MOL000906 | Wenjine | 47.93 | 0.27 | 0.30 | 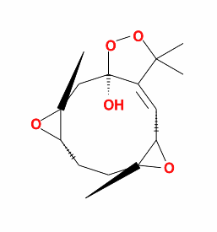 | *CR* |
| 31 | MOL000940 | Bisdemethoxycurcumin | 77.38 | 0.26 | 0.49 | 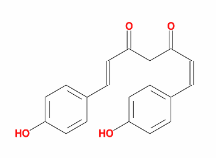 | *CR* |
| 32 | MOL001506 | Supraene | 33.55 | 0.42 | 2.08 | 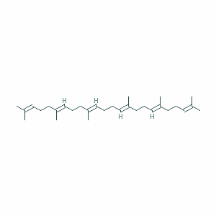 | *PR* |
| 33 | MOL001601 | 1,2,5,6-tetrahydrotanshinone | 38.75 | 0.36 | 0.96 | 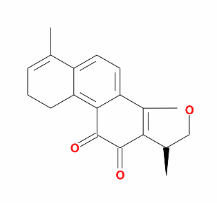 | *RS* |
| 34 | MOL001646 | 2,3-dimethoxy-6-methyanthraquinone | 34.86 | 0.26 | 0.75 | 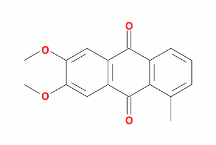 | *HDH* |
| 35 | MOL001659 | Poriferasterol | 43.83 | 0.76 | 1.44 | 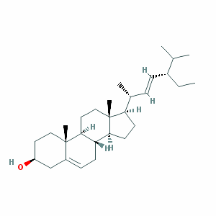 | *RS，HDH* |
| 36 | MOL001663 | (4aS,6aR,6aS,6bR,8aR,10R,12aR,14bS)-10-hydroxy-2,2,6a,6b,9,9,12a-heptamethyl-1,3,4,5,6,6a,7,8,8a,10,11,12,13,14b-tetradecahydropicene-4a-carboxylic acid | 32.03 | 0.76 | 0.61 | 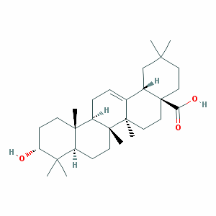 | *HDH* |
| 37 | MOL001670 | 2-methoxy-3-methyl-9,10-anthraquinone | 37.83 | 0.21 | 0.73 | 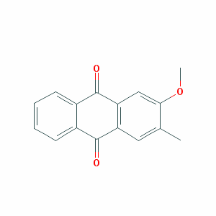 | *HDH* |
| 38 | MOL001689 | Acacetin | 34.97 | 0.24 | 0.67 | 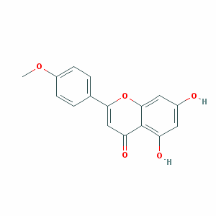 | *PR* |
| 39 | MOL001771 | Poriferast-5-en-3beta-ol | 36.91 | 0.75 | 1.45 | 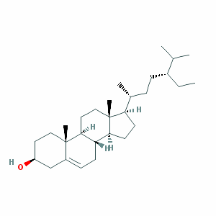 | *RS* |
| 40 | MOL001942 | Isoimperatorin | 45.46 | 0.23 | 0.97 | 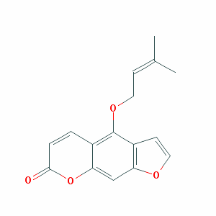 | *RS* |
| 41 | MOL002222 | Sugiol | 36.11 | 0.28 | 1.14 | 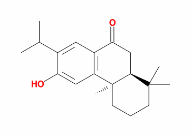 | *RS* |
| 42 | MOL002464 | 1-Monolinolein | 37.18 | 0.30 | 0.32 | 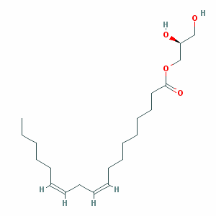 | *PR* |
| 43 | MOL002651 | Dehydrotanshinone II A | 43.76 | 0.40 | 1.02 | 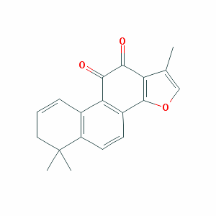 | *RS* |
| 44 | MOL006554 | Taraxerol | 38.40 | 0.77 | 1.37 | 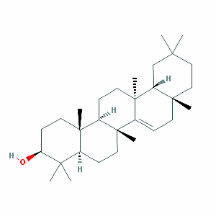 | *PR* |
| 45 | MOL006756 | Schottenol | 37.42 | 0.75 | 1.33 | 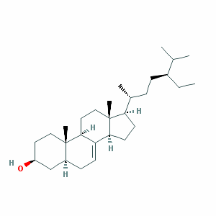 | *PR* |
| 46 | MOL006824 | α-amyrin | 39.51 | 0.76 | 1.37 | 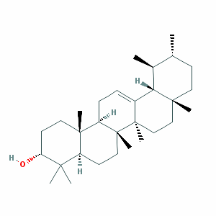 | *RS* |
| 47 | MOL007036 | 5,6-dihydroxy-7-isopropyl-1,1-dimethyl-2,3-dihydrophenanthren-4-one | 33.77 | 0.29 | 1.19 | 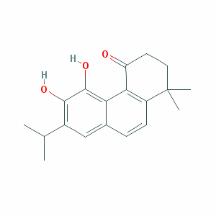 | *RS* |
| 48 | MOL007041 | 2-isopropyl-8-methylphenanthrene-3,4-dione | 40.86 | 0.23 | 1.23 | 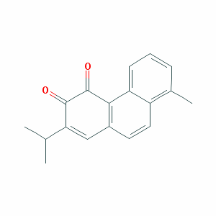 | *RS* |
| 49 | MOL007045 | 3α-hydroxytanshinoneⅡA | 44.93 | 0.44 | 0.53 | 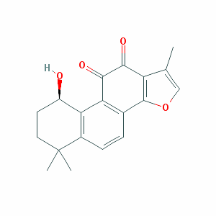 | *RS* |
| 50 | MOL007048 | (E)-3-[2-(3,4-dihydroxyphenyl)-7-hydroxy-benzofuran-4-yl]acrylic acid | 48.24 | 0.31 | 0.18 | 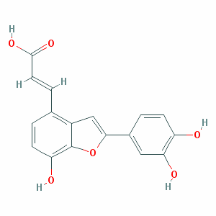 | *RS* |
| 51 | MOL007049 | 4-methylenemiltirone | 34.35 | 0.23 | 1.25 | 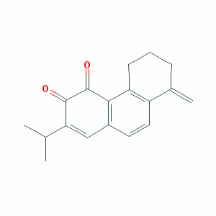 | *RS* |
| 52 | MOL007050 | 2-(4-hydroxy-3-methoxyphenyl)-5-(3-hydroxypropyl)-7-methoxy-3-benzofurancarboxaldehyde | 62.78 | 0.40 | 0.35 | 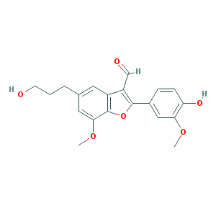 | *RS* |
| 53 | MOL007058 | Formyltanshinone | 73.44 | 0.42 | 0.54 | 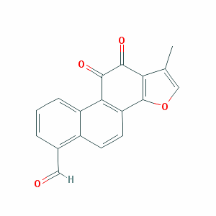 | *RS* |
| 54 | MOL007059 | 3-beta-Hydroxymethyllenetanshiquinone | 32.16 | 0.41 | 0.38 | 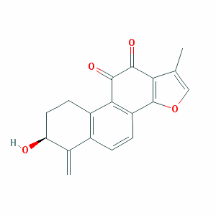 | *RS* |
| 55 | MOL007061 | Methylenetanshinquinone | 37.07 | 0.36 | 1.03 | 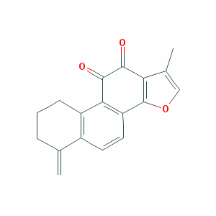 | *RS* |
| 56 | MOL007064 | Przewalskin B | 110.32 | 0.44 | 0.34 | 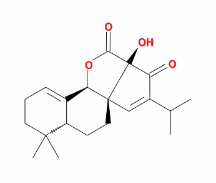 | *RS* |
| 57 | MOL007068 | Przewaquinone B | 62.24 | 0.41 | 0.39 | 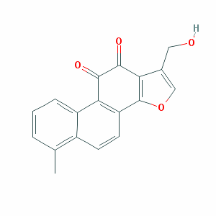 | *RS* |
| 58 | MOL007069 | Przewaquinone C | 55.74 | 0.40 | 0.42 | 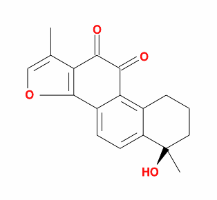 | *RS* |
| 59 | MOL007077 | Sclareol | 43.67 | 0.21 | 0.84 | 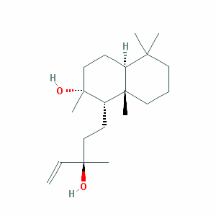 | *RS* |
| 60 | MOL007079 | Tanshinaldehyde | 52.47 | 0.45 | 0.57 | 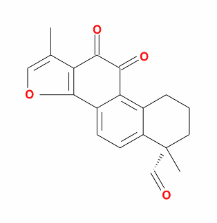 | *RS* |
| 61 | MOL007081 | Danshenol B | 57.95 | 0.56 | 0.53 | 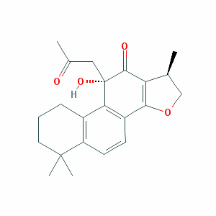 | *RS* |
| 62 | MOL007082 | Danshenol A | 56.97 | 0.52 | 0.33 | 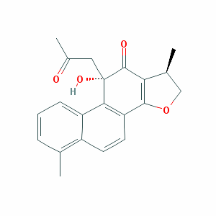 | *RS* |
| 63 | MOL007085 | Salvilenone | 30.38 | 0.38 | 1.46 | 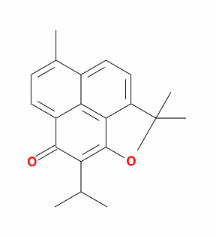 | *RS* |
| 64 | MOL007088 | Cryptotanshinone | 52.34 | 0.40 | 0.95 | 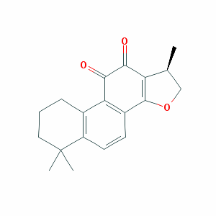 | *RS* |
| 65 | MOL007093 | Dan-shexinkum d | 38.88 | 0.55 | 0.67 | 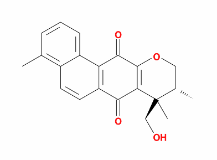 | *RS* |
| 66 | MOL007094 | Danshenspiroketallactone | 50.43 | 0.31 | 0.88 | 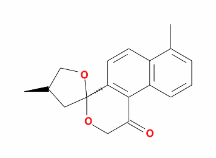 | *RS* |
| 67 | MOL007098 | Deoxyneocryptotanshinone | 49.40 | 0.29 | 0.85 | 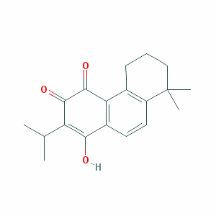 | *RS* |
| 68 | MOL007100 | Dihydrotanshinlactone | 38.68 | 0.32 | 1.26 | 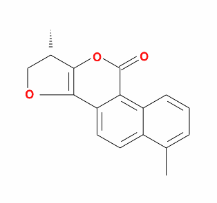 | *RS* |
| 69 | MOL007101 | DihydrotanshinoneⅠ | 45.04 | 0.36 | 0.95 | 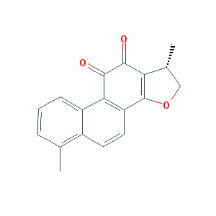 | *RS* |
| 70 | MOL007105 | Epidanshenspiroketallactone | 68.27 | 0.31 | 0.90 | 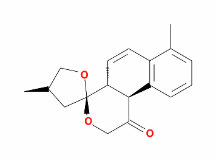 | *RS* |
| 71 | MOL007107 | C09092 | 36.07 | 0.25 | 1.63 | 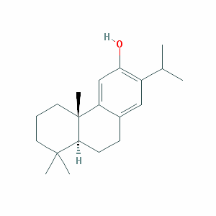 | *RS* |
| 72 | MOL007108 | Isocryptotanshi-none | 54.98 | 0.39 | 0.93 | 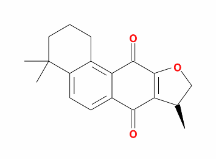 | *RS* |
| 73 | MOL007111 | Isotanshinone II | 49.92 | 0.40 | 1.03 | 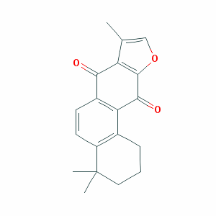 | *RS* |
| 74 | MOL007115 | Manool | 45.04 | 0.20 | 1.28 | 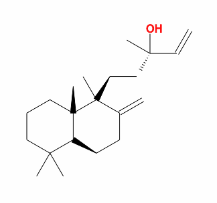 | *RS* |
| 75 | MOL007118 | Microstegiol | 39.61 | 0.28 | 1.05 | 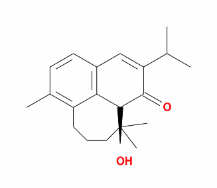 | *RS* |
| 76 | MOL007119 | Miltionone Ⅰ | 49.68 | 0.32 | 0.35 | 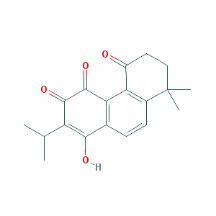 | *RS* |
| 77 | MOL007120 | Miltionone Ⅱ | 71.03 | 0.44 | 0.62 | 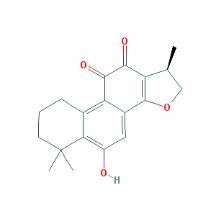 | *RS* |
| 78 | MOL007121 | Miltipolone | 36.56 | 0.37 | 0.50 | 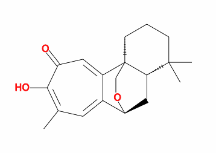 | *RS* |
| 79 | MOL007122 | Miltirone | 38.76 | 0.25 | 1.23 | 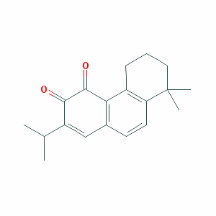 | *RS* |
| 80 | MOL007123 | Miltirone Ⅱ | 44.95 | 0.24 | 0.04 | 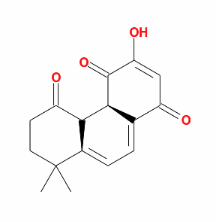 | *RS* |
| 81 | MOL007124 | Neocryptotanshinone Ⅱ | 39.46 | 0.23 | 0.76 | 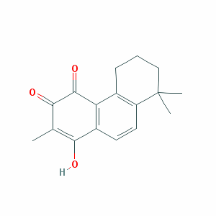 | *RS* |
| 82 | MOL007125 | Neocryptotanshinone | 52.49 | 0.32 | 0.35 | 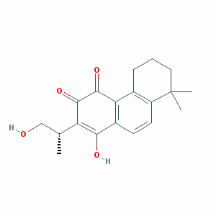 | *RS* |
| 83 | MOL007127 | 1-methyl-8,9-dihydro-7H-naphtho[5,6-g]benzofuran-6,10,11-trione | 34.72 | 0.37 | 0.50 | 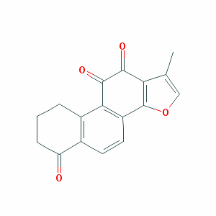 | *RS* |
| 84 | MOL007130 | Prolithospermic acid | 64.37 | 0.31 | 0.10 | 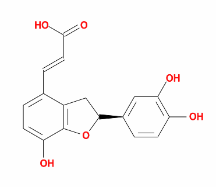 | *RS* |
| 85 | MOL007134 | Danshensu | 36.91 | 0.06 | -0.27 | 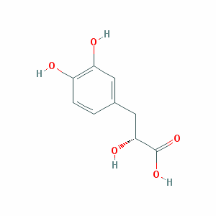 | *RS* |
| 86 | MOL007143 | Salvilenone Ⅰ | 32.43 | 0.23 | 1.13 | 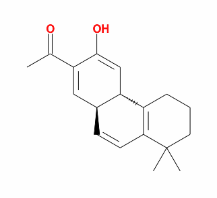 | *RS* |
| 87 | MOL007145 | Salviolone | 31.72 | 0.24 | 1.04 | 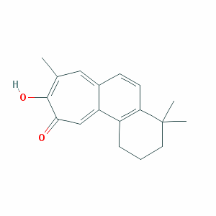 | *RS* |
| 88 | MOL007149 | NSC 122421 | 34.49 | 0.28 | 1.08 | 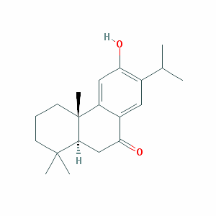 | *RS* |
| 89 | MOL007150 | (6S)-6-hydroxy-1-methyl-6-methylol-8,9-dihydro-7H-naphtho[8,7-g]benzofuran-10,11-quinone | 75.39 | 0.46 | 0.03 | 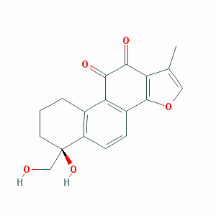 | *RS* |
| 90 | MOL007151 | Tanshindiol B | 42.67 | 0.45 | 0.05 | 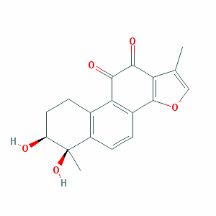 | *RS* |
| 91 | MOL007154 | Tanshinone Ⅱa | 49.89 | 0.40 | 1.05 | 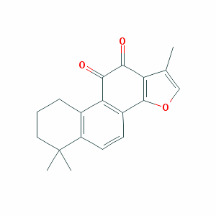 | *RS* |
| 92 | MOL007155 | (6S)-6-(hydroxymethyl)-1,6-dimethyl-8,9-dihydro-7H-naphtho[8,7-g]benzofuran-10,11-dione | 65.26 | 0.45 | 0.44 | 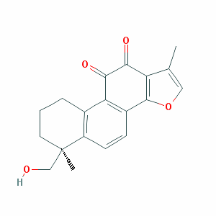 | *RS* |
| 93 | MOL007156 | Tanshinone Ⅵ | 45.64 | 0.30 | 0.48 | 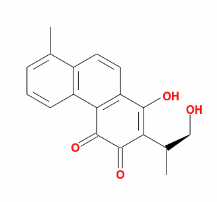 | *RS* |

**Table S2**

The information of GPL-related targets. By combining the compound targets of WPX and the disease related targets, 146 overlapping ones were selected as the key targets in the treatment of GPL.

| ID | UniProt | Protein names | Gene names | Organism |
| --- | --- | --- | --- | --- |
| T01 | Q9UNQ0 | ATP-binding cassette sub-family G member 2 | ABCG2 | Homo sapiens |
| T02 | P15309 | Prostatic acid phosphatase | ACPP | Homo sapiens |
| T03 | P00326 | Alcohol dehydrogenase 1C | ADH1C | Homo sapiens |
| T04 | P35869 | Aryl hydrocarbon receptor | AHR | Homo sapiens |
| T05 | P15121 | Aldose reductase | AKR1B1 | Homo sapiens |
| T06 | O60218 | Aldo-keto reductase family 1 member B10 | AKR1B10 | Homo sapiens |
| T07 | P31749 | RAC-alpha serine/threonine-protein kinase | AKT1 | Homo sapiens |
| T08 | P18054 | Arachidonate 12-lipoxygenase, 12S-type | ALOX12 | Homo sapiens |
| T09 | P09917 | Arachidonate 5-lipoxygenase | ALOX5 | Homo sapiens |
| T10 | P10275 | Androgen receptor | AR | Homo sapiens |
| T11 | Q07812 | Apoptosis regulator BAX | BAX | Homo sapiens |
| T12 | P10415 | Apoptosis regulator Bcl-2 | BCL2 | Homo sapiens |
| T13 | Q07817 | Bcl-2-like protein 1 | BCL2L1 | Homo sapiens |
| T14 | O15392 | Baculoviral IAP repeat-containing protein 5 | BIRC5 | Homo sapiens |
| T15 | P42574 | Caspase-3 | CASP3 | Homo sapiens |
| T16 | Q14790 | Caspase-8 | CASP8 | Homo sapiens |
| T17 | P55211 | Caspase-9 | CASP9 | Homo sapiens |
| T18 | Q03135 | Caveolin-1 | CAV1 | Homo sapiens |
| T19 | P13500 | C-C motif chemokine 2 | CCL2 | Homo sapiens |
| T20 | P20248 | Cyclin-A2 | CCNA2 | Homo sapiens |
| T21 | P14635 | G2/mitotic-specific cyclin-B1 | CCNB1 | Homo sapiens |
| T22 | P24385 | G1/S-specific cyclin-D1 | CCND1 | Homo sapiens |
| T23 | P06493 | Cell division control protein 2 homolog | CDK1 | Homo sapiens |
| T24 | P24941 | Cell division protein kinase 2 | CDK2 | Homo sapiens |
| T25 | P11802 | Cell division protein kinase 4 | CDK4 | Homo sapiens |
| T26 | P38936 | Cyclin-dependent kinase inhibitor 1 | CDKN1A | Homo sapiens |
| T27 | P42771 | Cyclin-dependent kinase inhibitor 2A, isoforms 1/2/3 | CDKN2A | Homo sapiens |
| T28 | O14757 | Serine/threonine-protein kinase Chk1 | CHEK1 | Homo sapiens |
| T29 | O96017 | Serine/threonine-protein kinase Chk2 | CHEK2 | Homo sapiens |
| T30 | O14493 | Claudin-4 | CLDN4 | Homo sapiens |
| T31 | P02452 | Collagen alpha-1(I) chain | COL1A1 | Homo sapiens |
| T32 | P02741 | C-reactive protein | CRP | Homo sapiens |
| T33 | P07339 | Cathepsin D | CTSD | Homo sapiens |
| T34 | P10145 | Interleukin-8 | CXCL8 | Homo sapiens |
| T35 | P11511 | Cytochrome P450 19A1 | CYP19A1 | Homo sapiens |
| T36 | P04798 | Cytochrome P450 1A1 | CYP1A1 | Homo sapiens |
| T37 | P05177 | Cytochrome P450 1A2 | CYP1A2 | Homo sapiens |
| T38 | Q16678 | Cytochrome P450 1B1 | CYP1B1 | Homo sapiens |
| T39 | P08684 | Cytochrome P450 3A4 | CYP3A4 | Homo sapiens |
| T40 | P27487 | Dipeptidyl peptidase IV | DPP4 | Homo sapiens |
| T41 | Q01094 | Transcription factor E2F2 | E2F1 | Homo sapiens |
| T42 | P01133 | Pro-epidermal growth factor | EGF | Homo sapiens |
| T43 | P00533 | Epidermal growth factor receptor | EGFR | Homo sapiens |
| T44 | P19419 | ETS domain-containing protein Elk-1 | ELK1 | Homo sapiens |
| T45 | P04626 | Receptor tyrosine-protein kinase erbB-2 | ERBB2 | Homo sapiens |
| T46 | P21860 | Receptor tyrosine-protein kinase erbB-3 | ERBB3 | Homo sapiens |
| T47 | P03372 | Estrogen receptor | ESR1 | Homo sapiens |
| T48 | Q92731 | Estrogen receptor beta | ESR2 | Homo sapiens |
| T49 | P13726 | Tissue factor | F3 | Homo sapiens |
| T50 | P48023 | Tumor necrosis factor ligand superfamily member 6 | FASLG | Homo sapiens |
| T51 | P49327 | Fatty acid synthase | FASN | Homo sapiens |
| T52 | P05230 | Fibroblast growth factor 1 | FGF1 | Homo sapiens |
| T53 | P09038 | Fibroblast growth factor 2 | FGF2 | Homo sapiens |
| T54 | P17948 | Vascular endothelial growth factor receptor 1 | FLT1 | Homo sapiens |
| T55 | P35916 | Vascular endothelial growth factor receptor 3 | FLT4 | Homo sapiens |
| T56 | P01100 | Proto-oncogene c-Fos | FOS | Homo sapiens |
| T57 | P17302 | Gap junction alpha-1 protein | GJA1 | Homo sapiens |
| T58 | P49841 | Glycogen synthase kinase-3 beta | GSK3B | Homo sapiens |
| T59 | P09488 | Glutathione S-transferase Mu 1 | GSTM1 | Homo sapiens |
| T60 | P09211 | Glutathione S-transferase P | GSTP1 | Homo sapiens |
| T61 | P14210 | Hepatocyte growth factor receptor | HGF | Homo sapiens |
| T62 | Q16665 | Hypoxia-inducible factor 1-alpha | HIF1A | Homo sapiens |
| T63 | P52789 | Hexokinase-2 | HK2 | Homo sapiens |
| T64 | P04035 | 3-hydroxy-3-methylglutaryl-coenzyme A reductase | HMGCR | Homo sapiens |
| T65 | P09601 | Heme oxygenase 1 | HMOX1 | Homo sapiens |
| T66 | Q9Y251 | Heparanase 8 kDa subunit | HPSE | Homo sapiens |
| T67 | P07900 | Heat shock protein HSP 90 | HSP90AA1 | Homo sapiens |
| T68 | P11021 | 78 kDa glucose-regulated protein | HSPA5 | Homo sapiens |
| T69 | P04792 | Heat shock protein beta-1 | HSPB1 | Homo sapiens |
| T70 | P05362 | Intercellular adhesion molecule 1 | ICAM1 | Homo sapiens |
| T71 | P01579 | Interferon gamma | IFNG | Homo sapiens |
| T72 | P01344 | Insulin-like growth factor II | IGF2 | Homo sapiens |
| T73 | P17936 | Insulin-like growth factor-binding protein 3 | IGFBP3 | Homo sapiens |
| T74 | O14920 | Inhibitor of nuclear factor kappa-B kinase subunit beta | IKBKB | Homo sapiens |
| T75 | P22301 | Interleukin-10 | IL10 | Homo sapiens |
| T76 | P01584 | Interleukin-1 beta | IL1B | Homo sapiens |
| T77 | P60568 | Interleukin-2 | IL2 | Homo sapiens |
| T78 | P05112 | Interleukin-4 | IL4 | Homo sapiens |
| T79 | P05231 | Interleukin-6 | IL6 | Homo sapiens |
| T80 | P06213 | Insulin receptor | INSR | Homo sapiens |
| T81 | P10914 | Interferon regulatory factor 1 | IRF1 | Homo sapiens |
| T82 | P05412 | Transcription factor AP-1 | JUN | Homo sapiens |
| T83 | Q12809 | Potassium voltage-gated channel subfamily H member 2 | KCNH2 | Homo sapiens |
| T84 | P35968 | Vascular endothelial growth factor receptor 2 | KDR | Homo sapiens |
| T85 | P09960 | Leukotriene A-4 hydrolase | LTA4H | Homo sapiens |
| T86 | P28482 | Mitogen-activated protein kinase 1 | MAPK1 | Homo sapiens |
| T87 | Q16539 | Mitogen-activated protein kinase 14 | MAPK14 | Homo sapiens |
| T88 | P45983 | Mitogen-activated protein kinase 8 | MAPK8 | Homo sapiens |
| T89 | Q07820 | Induced myeloid leukemia cell differentiation protein Mcl-1 | MCL1 | Homo sapiens |
| T90 | Q00987 | E3 ubiquitin-protein ligase Mdm2 | MDM2 | Homo sapiens |
| T91 | O43451 | Maltase-glucoamylase, intestinal | MGAM | Homo sapiens |
| T92 | P03956 | Interstitial collagenase | MMP1 | Homo sapiens |
| T93 | P08253 | 72 kDa type IV collagenase | MMP2 | Homo sapiens |
| T94 | P08254 | Stromelysin-1 | MMP3 | Homo sapiens |
| T95 | P14780 | Matrix metalloproteinase-9 | MMP9 | Homo sapiens |
| T96 | P05164 | Myeloperoxidase | MPO | Homo sapiens |
| T97 | P01106 | Myc proto-oncogene protein | MYC | Homo sapiens |
| T98 | P21359 | Neurofibromin | NF1 | Homo sapiens |
| T99 | Q16236 | Nuclear factor erythroid 2-related factor 2 | NFE2L2 | Homo sapiens |
| T100 | Q99801 | Homeobox protein Nkx-3.1 | NKX3-1 | Homo sapiens |
| T101 | P35228 | Nitric oxide synthase, inducible | NOS2 | Homo sapiens |
| T102 | P15559 | NAD(P)H dehydrogenase [quinone] 1 | NQO1 | Homo sapiens |
| T103 | O75469 | Nuclear receptor subfamily 1 group I member 2 | NR1I2 | Homo sapiens |
| T104 | P11926 | Ornithine decarboxylase | ODC1 | Homo sapiens |
| T105 | P09874 | Poly [ADP-ribose] polymerase 1 | PARP1 | Homo sapiens |
| T106 | P12004 | Proliferating cell nuclear antigen | PCNA | Homo sapiens |
| T107 | P06401 | Progesterone receptor | PGR | Homo sapiens |
| T108 | P48736 | Phosphatidylinositol-4,5-bisphosphate 3-kinase catalytic subunit, gamma isoform | PIK3CG | Homo sapiens |
| T109 | P11309 | Proto-oncogene serine/threonine-protein kinase Pim-1 | PIM1 | Homo sapiens |
| T110 | P00749 | Urokinase-type plasminogen activator | PLAU | Homo sapiens |
| T111 | Q07869 | Peroxisome proliferator-activated receptor alpha | PPARA | Homo sapiens |
| T112 | Q03181 | Peroxisome proliferator-activated receptor delta | PPARD | Homo sapiens |
| T113 | P37231 | Peroxisome proliferator-activated receptor gamma | PPARG | Homo sapiens |
| T114 | P05771 | Protein kinase C beta type | PRKCB | Homo sapiens |
| T115 | P07477 | Trypsin-1 | PRSS1 | Homo sapiens |
| T116 | P60484 | Phosphatidylinositol-3,4,5-trisphosphate 3-phosphatase and dual-specificity protein phosphatase PTEN | PTEN | Homo sapiens |
| T117 | P43115 | Prostaglandin E2 receptor EP3 subtype | PTGER3 | Homo sapiens |
| T118 | P23219 | Prostaglandin G/H synthase 1 | PTGS1 | Homo sapiens |
| T119 | P35354 | Prostaglandin G/H synthase 2 | PTGS2 | Homo sapiens |
| T120 | P04049 | RAF proto-oncogene serine/threonine-protein kinase | RAF1 | Homo sapiens |
| T121 | Q9NS23 | Ras association domain-containing protein 1 | RASSF1 | Homo sapiens |
| T122 | P06400 | Retinoblastoma-associated protein | RB1 | Homo sapiens |
| T123 | Q04206 | Transcription factor p65 | RELA | Homo sapiens |
| T124 | Q13950 | Runt-related transcription factor 2 | RUNX2 | Homo sapiens |
| T125 | P19793 | Retinoic acid receptor RXR-alpha | RXRA | Homo sapiens |
| T126 | P28702 | Retinoic acid receptor RXR-beta | RXRB | Homo sapiens |
| T127 | P05121 | Plasminogen activator inhibitor 1 | SERPINE1 | Homo sapiens |
| T128 | Q96EB6 | NAD-dependent deacetylase sirtuin-1 | SIRT1 | Homo sapiens |
| T129 | P03973 | Antileukoproteinase | SLPI | Homo sapiens |
| T130 | P00441 | Superoxide dismutase [Cu-Zn] | SOD1 | Homo sapiens |
| T131 | P10451 | Osteopontin | SPP1 | Homo sapiens |
| T132 | P12931 | Proto-oncogene tyrosine-protein kinase Src | SRC | Homo sapiens |
| T133 | P42224 | Signal transducer and activator of transcription 1-alpha/beta | STAT1 | Homo sapiens |
| T134 | P40763 | Signal transducer and activator of transcription 3 | STAT3 | Homo sapiens |
| T135 | P01137 | Transforming growth factor beta-1 | TGFB1 | Homo sapiens |
| T136 | P07204 | Thrombomodulin | THBD | Homo sapiens |
| T137 | P01033 | Metalloproteinase inhibitor 1 | TIMP1 | Homo sapiens |
| T138 | Q9NR96 | Toll-like receptor 9 | TLR9 | Homo sapiens |
| T139 | P01375 | Tumor necrosis factor | TNF | Homo sapiens |
| T140 | P11387 | DNA topoisomerase 1 | TOP1 | Homo sapiens |
| T141 | P11388 | DNA topoisomerase 2-alpha | TOP2A | Homo sapiens |
| T142 | P04637 | Cellular tumor antigen p53 | TP53 | Homo sapiens |
| T143 | P15692 | Vascular endothelial growth factor A | VEGFA | Homo sapiens |
| T144 | P47989 | Xanthine dehydrogenase/oxidase | XDH | Homo sapiens |
| T145 | P98170 | Baculoviral IAP repeat-containing protein 4 | XIAP | Homo sapiens |
| T146 | P07947 | Tyrosine-protein kinase Yes | YES1 | Homo sapiens |

**Table S3**

The GO terms of therapy target genes and their corresponding Count, *P*Value, FDR. Through the GO enrichment of the key targets, 26 top GO terms were obtained which indicate that large numbers of targets involved in the process of tumorigenesis.

| **GO ID** | **Term** | **Count** | ***P*Value** | **FDR** |
| --- | --- | --- | --- | --- |
| GO:0045944 | positive regulation of transcription from RNA polymerase II promoter | 48 | 4.35E-23 | 7.66E-20 |
| GO:0043066 | negative regulation of apoptotic process | 39 | 5.03E-27 | 8.86E-24 |
| GO:0045893 | positive regulation of transcription, DNA-templated | 39 | 4.54E-25 | 7.99E-22 |
| GO:0008284 | positive regulation of cell proliferation | 35 | 2.99E-22 | 5.27E-19 |
| GO:0007165 | signal transduction | 31 | 4.88E-08 | 8.58E-05 |
| GO:0042493 | response to drug | 30 | 3.01E-22 | 5.30E-19 |
| GO:0010628 | positive regulation of gene expression | 27 | 1.96E-20 | 3.46E-17 |
| GO:0006915 | apoptotic process | 26 | 1.70E-11 | 2.99E-08 |
| GO:0000122 | negative regulation of transcription from RNA polymerase II promoter | 23 | 2.39E-07 | 4.21E-04 |
| GO:0007568 | aging | 20 | 2.28E-16 | 3.89E-13 |
| GO:0043065 | positive regulation of apoptotic process | 20 | 1.28E-11 | 2.25E-08 |
| GO:0008285 | negative regulation of cell proliferation | 20 | 1.44E-09 | 2.53E-06 |
| GO:0045892 | negative regulation of transcription, DNA-templated | 20 | 6.02E-08 | 1.06E-04 |
| GO:0001525 | angiogenesis | 19 | 7.81E-13 | 1.37E-09 |
| GO:0006954 | inflammatory response | 19 | 4.72E-09 | 8.31E-06 |
| GO:0071456 | cellular response to hypoxia | 18 | 5.33E-18 | 9.37E-15 |
| GO:0045766 | positive regulation of angiogenesis | 18 | 1.29E-16 | 2.00E-13 |
| GO:0008283 | cell proliferation | 18 | 1.81E-08 | 3.19E-05 |
| GO:0006468 | protein phosphorylation | 17 | 2.10E-06 | 0.003686 |
| GO:0001934 | positive regulation of protein phosphorylation | 16 | 2.71E-13 | 4.77E-10 |
| GO:0001666 | response to hypoxia | 16 | 2.33E-11 | 4.09E-08 |
| GO:0000165 | MAPK cascade | 16 | 8.27E-09 | 1.45E-05 |
| GO:0032355 | response to estradiol | 15 | 4.10E-14 | 7.21E-11 |
| GO:0071222 | cellular response to lipopolysaccharide | 15 | 8.95E-13 | 1.58E-09 |
| GO:0070374 | positive regulation of ERK1 and ERK2 cascade | 15 | 3.52E-10 | 6.19E-07 |
| GO:0006974 | cellular response to DNA damage stimulus | 15 | 3.38E-09 | 5.95E-06 |

**Table S4**

The KEGG Pathways of therapy target genes and their corresponding Count, *P*Value and FDR. Through the KEGG enrichment of the key targets, 21 remarkably enriched pathways which involved in cell proliferation, apoptosis and inflammation were obtained.

| **Term** | **Pathways** | **Count** | ***P*value** | **FDR** |
| --- | --- | --- | --- | --- |
| hsa05200 | Pathways in cancer | 58 | 5.71E-36 | 7.20E-33 |
| hsa04151 | PI3K-Akt signaling pathway | 37 | 3.03E-17 | 3.82E-14 |
| hsa04010 | MAPK signaling pathway | 24 | 7.32E-10 | 9.24E-07 |
| hsa04014 | Ras signaling pathway | 23 | 4.16E-10 | 5.25E-07 |
| hsa04068 | FoxO signaling pathway | 22 | 9.91E-14 | 1.25E-10 |
| hsa04066 | HIF-1 signaling pathway | 21 | 1.99E-15 | 2.52E-12 |
| hsa04668 | TNF signaling pathway | 19 | 1.60E-12 | 2.02E-09 |
| hsa04115 | p53 signaling pathway | 18 | 6.05E-15 | 7.56E-12 |
| hsa04110 | Cell cycle | 18 | 2.38E-10 | 3.00E-07 |
| hsa05202 | Transcriptional misregulation in cancer | 18 | 2.72E-08 | 3.43E-05 |
| hsa04060 | Cytokine-cytokine receptor interaction | 18 | 2.52E-06 | 0.003174625 |
| hsa04620 | Toll-like receptor signaling pathway | 17 | 1.91E-10 | 2.41E-07 |
| hsa04015 | Rap1 signaling pathway | 17 | 3.43E-06 | 0.004325218 |
| hsa04012 | ErbB signaling pathway | 16 | 1.04E-10 | 1.31E-07 |
| hsa04660 | T cell receptor signaling pathway | 16 | 1.23E-09 | 1.55E-06 |
| hsa04210 | Apoptosis | 14 | 1.50E-10 | 1.89E-07 |
| hsa04915 | Estrogen signaling pathway | 13 | 4.83E-07 | 6.09E-04 |
| hsa04621 | NOD-like receptor signaling pathway | 12 | 7.07E-09 | 8.92E-06 |
| hsa04370 | VEGF signaling pathway | 12 | 2.23E-08 | 2.82E-05 |
| hsa05230 | Central carbon metabolism in cancer | 11 | 4.11E-07 | 5.19E-04 |
| hsa04064 | NF-kappa B signaling pathway | 11 | 7.37E-06 | 0.009296675 |
